# A Comment on Qi *et al*.: An Estimation of Radiobiological Parameters for Head-and-Neck and the Clinical Implications. *Cancers*, 2012, *4*, 566-580

**DOI:** 10.3390/cancers4041225

**Published:** 2012-11-22

**Authors:** Jack F. Fowler

**Affiliations:** Emeritus of Departments of Human Oncology and Medical Physics, School of Medicine and Public Health, University of Wisconsin, Madison, WI, USA; Present address: 150 Lambeth Rd., London SE1 7DF, UK; E-Mail: jackfowlersbox@gmail.com

**Keywords:** recovery, repair, half-times

## Abstract

Important results were shown as cell survival points in the two panels of the figure which is reproduced in this Comment letter. A curve was fitted assuming the mono-exponential recovery half-time of 17 ± 21 minutes. The wide error limits indicate that this fit is not very good, but the notable feature of both panels is that the last four points are clearly continuing to rise, above the “fitted” curve. This indicates that there is a second, slower, component of repair or recovery and this Comment explores constructively the implications of that additional discovery.

## 1. Introduction

**Figure 1:**
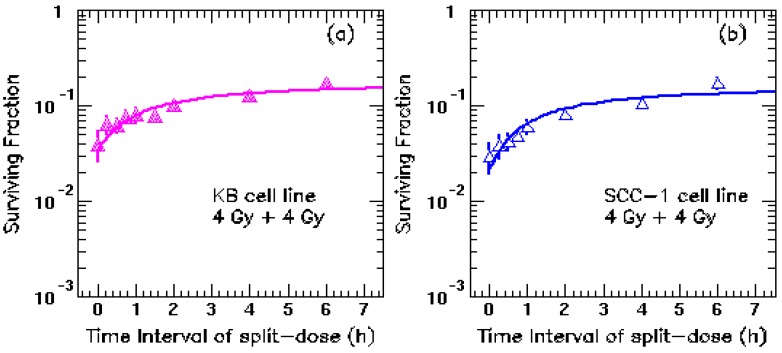
Cell survival points from dishes each seeded with 3,000 cells, irradiated at intervals of 0 to to 6 hours as shown with two doses of 8 Gy at 6 MV with full build-up at peak depth in medium, at the stated intervals up to 6 hours; (**a**) cells from a primary epidermal cacrinoma and (**b**) cells from a primary squamous cell carcinoma of the retromolar trigone (obtained as in [1]), cultured for three weeks then counted for colonies exceeding 50 cells. The fitted curves are for 18 ± 21 and 16 ± 25 minutes respectively. Reproduced from [1].

The interesting paper by Qi *et al*. [1] contains an additional point of interest other than those the authors show and discuss. They correctly describe a half-time of about 17 min, based on two human Head-and-Neck Cancer cell lines, but both their two experimental result panels, Figure 1a,b clearly shows that there is also a longer (slower) component of repair than the 17 min one that they mention.

## 2. Experimental Section

These continuously rising curves are obvious from the fact that neither of the curves that they show [1] flatten out sufficiently to pass through the final four points of their Figure 1a or of Figure 1b. Intervals used were 0, 15, 30, 45, 60 min and 2, 4, and 6 h.

These discrepancies between the curves shown in each of the panels are obvious by eye and are not matched by the curves drawn through them in the panels’ final four points.

## 3. Results

The fact that recovery is far from complete even after two periods of 3 h is emphasised by drawing best straight lines through the final four points that are shown in each of the two panels of Figure 1a,b. This is a familiar problem for those accustomed to analysing multiple exponential components, but it seems that Qi *et al*. might not be familiar with the arguments that went on between many groups of animal experimenters between the late 1980s and the early 2000s about the existence of two repair half-times in the normal tissues of animals [2] and also in human patients [3]. I didn’t myself believe them at first, until I did some animal experiments and also found them, as listed in detail in [2].

The mathematics are simple—exactly as in the analyses of a mixture of many radionuclides with their different rates of decay. One does not necessarily expect only one nuclide with a single half-time, and there are many reasons to expect biological recovery rates to occur with a large range of rates instead of a single exponential rate, even in one tissue [2]. Experiments using only short intervals will see only short half-times, while experiments using only long intervals will detect only long half-times [4]. The present experiments cover a wide range of intervals and thus can detect two half-times if there are two present. A single half-time is an over-smplified expectation in biology, and even two exponential exponents is probably also an oversimplification. We may be too fixed on the concept of mono-exponential recovery only—it is convenient but unlikely to be true in the complicated series of processes that occur in biological recovery.

The more recent large analysis by Ling *et al*. [5] accepts that two half-times are present in normal tissues but failed to find evidence for them in tumor cells. This is where the present results from Figure 1a,b are of particular interest. Bentzen *et al*. [4] missed entirely the shorter components, similar to the ~17 min analysed by Qi *et al*. here [1], because they had only 6 h and 18 h intervals in their patients treated three times a day in the CHART trials [4]; they saw nothing shorter than about 4 h.

Qi *et al*. are interested only in the effects of “long fraction” durations in many minutes, so their main conclusions on that subject [1] are not altered. However, the right-hand ends of their Figure 2 should be lifted a few percent, at 6 or 7 h, to allow for 40–60% of their 3.5–4 h recovery to take place at the slower rate of T½ ~4 h, a small correction, but the presence of this longer component will alter the overnight accumulated total doses for any Head-and-Neck schedules that utilise two fractions a day and should then be taken into account within any 5-day week. Equally, the 17-min component emphasisd in this paper [1] helps to keep such accumulated total doses from being excessive.

## 4. Discussion

It is unlikely that the two presently identified half-times of 17 min and 4 h [1] are so similar to the two values of 10–20 min and 4 h found previously [2,4] just by coincidence. It now appears that the two approaches are converging to give a more general description of recovery in animal tissues, with two components in the current approximation. The existence of the two components has been known for some years, but surprisingly ignored—and sometimes vociferously denied—but the multi-component nature of recovery after irradiation now appears to be reaching agreement as one component of 15–20 min and the other of 3–5 h The presence of both components was indeed summarised and utilized by Fowler, Welsh and Howard in 2004 for late-responding normal tissues [2], but it is the first time that it has been reported for tumor cells, and indeed has often been denied in tumors [3], as has the shorter component by Bentzen and Yarnold [6] in another treatment.

## 5. Conclusions

The present clear experimental results in the last four points of Figure 1a,b here [1] suggest that bi-exponential repair is indeed possible in some tumor cells and could be common among many types of cells. Further evidence should be sought to find out how often recovery in this pattern occurs, possibly in both tumor and normal cells.
